# A Major Facilitator Superfamily Peptide Transporter From *Fusarium oxysporum* Influences Bioethanol Production From Lignocellulosic Material

**DOI:** 10.3389/fmicb.2019.00295

**Published:** 2019-02-26

**Authors:** Brian Nugent, Shahin S. Ali, Ewen Mullins, Fiona M. Doohan

**Affiliations:** ^1^Molecular Plant-Microbe Interactions Laboratory, School of Biology and Environmental Science, University College Dublin, Dublin, Ireland; ^2^Department of Crop Science, Teagasc Research Centre, Carlow, Ireland

**Keywords:** bioethanol, *Fusarium*, peptide transporter, consolidated bioprocessing, lignocellulose

## Abstract

*Fusarium oxysporum* is a leading microbial agent in the emerging consolidated bioprocessing (CBP) industry owing to its capability to infiltrate the plant’s lignin barrier and degrade complex carbohydrates to value-added chemicals such as bioethanol in a single step. Membrane transport of nutrients is a key factor in successful microbial colonization of host tissue. This study assessed the impact of a peptide transporter on *F. oxysporum’s* ability to convert lignocellulosic straw to ethanol. We characterized a novel *F. oxysporum* peptide transporter (*FoPTR2*) of the dipeptide/tripeptide transporter (PTR) class. *FoPTR2* represents a novel transporter with high homology to the *Trichoderma sp.* peptide transporters *ThPTR2* and *TrEST-AO793*. Its expression level was highly activated in nitrogen-poor environments, which is a characteristic of PTR class peptide transporters. Overexpression and post-translational gene silencing of the *FoPTR2* in *F. oxysporum* affected the peptide transport capacity and ethanol yielded from a both a wheat straw/bran mix and glucose. Thus, we conclude that it *FoPTR2* plays a role in the nutrient acquisition system of *F. oxysporum* which serves to not only enhance fungal fitness but also CBP efficacy.

## Introduction

Industry and academics alike continually seek to increase the efficacy and reduce the processing costs of bioethanol production from lignocellulosic materials. Most research efforts are focused on improving inadequacies in the pretreatment process or the costly enzymatic hydrolysis step. Over the last decade, a lot of scientific attention has focused on consolidated bioprocessing (CBP), a process whereby substrate saccharification is conducted by one or more microbes within a single step process (Xu et al., 2009; Ali et al., 2016; Lynd et al., 2017). *Fusarium oxysporum* is a plant pathogen which has the ability to infiltrate the plant’s lignin barrier and degrade complex carbohydrates to value-added chemicals, and these traits have made it a leading microbial agent in the emerging CBP industry (Panagiotou et al., 2005, 2011; Xiros and Christakopoulos, 2009; Ali et al., 2014, 2016).

Previous work by Ali et al. (2014) identified a large number of *F. oxysporum* genes up-regulated during bioethanol production from lignocellulosic material. One such gene encoded a putative peptide transporter (*FoPTR2*) protein. Peptide transporters transport small molecular-weight peptides (2–6 amino acids) across cellular membranes, against a concentration gradient, into the cell (Steiner et al., 1995). The ability of organisms to execute carrier-mediated transport of small peptides is ubiquitous (Hauser et al., 2001). Transported peptides are then promptly hydrolyzed to amino acids by intracellular peptidases and used for protein synthesis or as alternative nitrogen and carbon sources (Vieira et al., 2013). Membrane transport of nutrients is a key factor in successful colonization during host-pathogen interactions (Divon et al., 2006; Divon and Fluhr, 2007). In addition, peptide transport has also been shown to assist pathogens in evading the host immune response system (Parra-Lopez et al., 1993).

Peptide transporters can be categorized into three super-families: (i) the ATP-binding cassette (ABC family); (ii) the oligopeptide (OPT) transporter family; (iii) and the peptide transporter (PTR/POT) family (Steiner et al., 1995; Pao et al., 1998; Reddy et al., 2012). Fungi possess mainly OPT and PTR/POT that allow them to utilize peptides as nutrients (Dunkel et al., 2013). Owing to a proposed adaptable coupling mechanism (Parker et al., 2014), many eukaryotic and prokaryotic members of the PTR/POT family have the versatility to symport a wide spectrum of nitrogen-containing substrates that include peptides, amino acids and nitrate (Williams and Miller, 2001; Chiang et al., 2004). It is believed that the PTR/POT system in cells is governed by the nitrogen catabolite repression (NCR) system (Perry et al., 1993). Coupled with this, conflicting evidence on the regulation of *PTR* genes by carbon catabolite repression (CCR) has also been reported (Vizcaíno et al., 2006; Vieira et al., 2013).

The purpose of this study was to characterize the *FoPTR2* gene up-regulated in *F. oxysporum* during bioethanol production from lignocellulosic material and to determine if the encoded protein impacts upon the ability of *F. oxysporum* to convert this substrate to bioethanol. The *FoPTR2* gene was overexpressed and post-translationally silenced in *F. oxysporum* strain 11C. The impact of gene overexpression and post-translational silencing on the fungal CBP capacity was determined. Gene expression studies investigated the impact of nitrogen and carbon sources on the regulation of *FoPTR2* transcription. On the basis of these studies, we discuss the potential contribution of peptide transporters to the CBP capacity of *F. oxysporum.*

## Materials and Methods

### Origin and Maintenance of Fungi

*Fusarium oxysporum* strains 11C (IMI501118) and 7E (IMI501116) used in this research were collected from diverse locations throughout Ireland (Ali et al., 2012). Fungal isolates were added to 30% glycerol (vv^-1^), flash-frozen using liquid nitrogen and a stock culture was stored at –70°C. Fungi were sub-cultured onto potato dextrose agar (PDA; Difco, United Kingdom) plates and were grown at 25°C for 5 days (Ali et al., 2012).

### Solid-State Cultivation (SSC) on Straw/Bran

Bioethanol production and gene expression studies were examined via independent solid-state cultivation experiments, as previously optimized by Ali et al. (2012). In brief, the solid state medium comprised minimal media (pH 5) containing 20% (wv^-1^) of a 10:1 wheat straw:bran mix as the carbon source. *F. oxysporum* conidia were generated in mung bean broth, as previously described (Brennan et al., 2005) and re-suspended into minimal medium (pH 5) (Mishra et al., 1984) to a final concentration of 10^6^ conidia ml^-1^. Medium was inoculated with 4 ml of fungal conidial suspension (minimal medium was added to negative controls) and the flasks were then sealed with either non-absorbent cotton (aerobic conditions) or cork and parafilm (oxygen-limited conditions) and incubated at 25°C in the dark. For the bioethanol production experiment, fungal mycelium was cultured under aerobic conditions for 96 h, which facilitated biomass and saccharolytic enzyme production, followed by an oxygen-limiting period of 96 h to facilitate fermentation. For the gene expression experiment, fungal mycelium was cultured under aerobic conditions for 24–94 h prior to RNA extraction. Each experiment comprised two biological replicates, each of which included three flasks per fungal strain/mutant.

### Hexose and Pentose Sugar Fermentation

Shake flask cultivation under oxygen-limiting conditions was used to assess the competency of wild type and mutant strains of *F. oxysporum* to ferment glucose and other pentose sugars, as previously described by Ali et al. (2013). In brief, minimal media (30 ml in 100 ml Erlenmeyer flasks) described by Leung et al. (1995), supplemented with 1% wv^-1^ carbon (glucose/cellulose/xylose) was inoculated with 4 ml of fungal conidia (10^6^ ml^-1^); negative controls were supplemented with 4 ml of minimal medium. Flasks were plugged with sterile cotton wool and incubated at 30°C, 150 rpm for 24 h (aerobic growth phase). Thereafter, flasks were plugged with cork, secured using parafilm, and incubated at 50 rpm, 30°C for 24–192 h prior to bioethanol measurement. The experiment comprised two biological replicates, each of which included three flasks per fungal strain/mutant.

### Estimation of Bioethanol and Fungal Biomass

For bioethanol estimation assays, samples were harvested as previously specified by Ali et al. (2012) and the bioethanol content was determined using the QuantiChromTM Ethanol Assay Kit (DIET-500; BioAssay Systems, United States). The fungal biomass (dry weight) in the solid portion of cultures was quantified as previously specified by Ali et al. (2012).

### Nitrogen and Glucose Experiment

Gene expression studies were used to determine the influence of the nitrogen and glucose concentration in aerobic shake flask cultures on the expression of *FoPTR2*. The minimal medium described by Penttila et al. (1987) was used (30 ml in 100 ml Erlenmeyer flasks) and ammonium sulfate and glucose concentrations were manipulated to mimic the following nitrogen starvation and glucose rich/limited conditions:(i) 2% glucose and 0.005% NH_4_SO_4_, (ii) 2% glucose and 0.05% NH_4_SO_4,_ (iii) 0% glucose and 0.5% NH_4_SO_4,_ (iv) 10% glucose and 0.5% NH_4_SO_4,_ (v) 2% glucose and 0.5% NH_4_SO_4_ (control). Flasks were inoculated with 4 ml of fungal conidia (10^6^ ml^-1^); negative controls were supplemented with 4 ml of minimal medium. The flasks were plugged with cotton wool, covered with aluminum foil and incubated at 150 rpm, 25°C for 4, 9 or 24h. Fungal mycelia was harvested at each time point by centrifugation and was washed twice with sterile distilled water. Mycelium was then flash frozen in liquid nitrogen and freeze-dried prior to RNA extraction. This experiment comprised three biological replicates, each including three flasks per medium per time point per treatment.

### RNA Isolation

Freeze-dried mycelial samples were homogenized in a mixer mill (Tissue Lyser II, Netherlands) at 30 Hz for 1 min with two 2.3 mm steel beads. RNA was isolated and quality-checked as previously described by Ali et al. (2012).

### Real Time RT-PCR Analysis

The real time qRT-PCR of the target gene and housekeeping gene was performed as described previously (Ali et al., 2014). The *F. oxysporum* β-tubulin gene was used as housekeeping gene for normalization of real-time RT-PCR data (Ali et al., 2013). See data sheet, Supplementary Table S1 for *FoPTR2*- and β-*tub*-specific primers. PCR reactions and data analysis were conducted as previously described by Ali et al. (2014). qRT-PCR was conducted twice for each sample and the average result used for data analysis.

### Rapid Amplification of cDNA Ends (RACE) and *in silico* Bioinformatic Analysis

5’- and 3’-RACE of *FoPTR2* gene from *F. oxysporum* strain 11C was conducted as previously described by Ali et al. (2013). See data sheet, Supplementary Table S1 for gene-specific primers RACE-*FoPtr2*-MF/MR. RACE products were gel-purified, cloned into the pGEM-T Easy vector (Promega, United States) and sequenced (Macrogen, Korea). The NCBI ORF finder^1^ was used to determine the ORF sequence. The presence of conserved domains in the *FoPTR2* amino acid sequence was assessed using the NCBI conserved domain database^2^. Transmembrane domain analysis was conducted using the TMHMM Server version 2.0^3^. The putative crystal structure was generated using SWISS-MODEL Workspace^4^. The peptide transporter sequences from *F. oxysporum* strain 4287 were obtained from the GenBank (bioproject PRJNA18813). An additional 37 peptide transporter proteins from fungi, mammals, plants and bacteria were obtained from the NCBI database. Protein sequences were aligned using the Clustal Omega tool^5^ and their evolutionary relationship was inferred using the maximum-likelihood algorithm with bootstrap (1000 replicates). Evolutionary analyses were carried out in MEGA6 (Tamura et al., 2013).

### Construction of the RNA Silencing Vector

*FoPTR2* silencing mutants of *F. oxysporum* strain 11C were generated using the pSilent-1 vector (Nakayashiki et al., 2005) (See data sheet, Supplementary Figure S1), A 415 bp fragment of the *FoPTR2* gene was inserted into each of the two multiple cloning sites in the sense and antisense direction (see data sheet, Supplementary Figure S1 and Supplementary Table S1 for primer information). The PCR amplification and vector construction were carried out as described by Ali et al. (2013). To check the correct alignment of sense and antisense segments, partial sequencing of the plasmid was conducted using the primers Ai-F/R (see data sheet, Supplementary Table S1).

### Construction of the Overexpression Vector

*FoPTR2* overexpression mutants of *F. oxysporum* strain 11C were generated using the pBARGPE1 vector (Pall and Brunelli, 1993) The PCR amplification and ligation of the full length *FoPTR2* gene into the pBARGPE1 vector was carried out as previously described by Ali et al. (2013). See data sheet, Supplementary Figure S2 and Supplementary Table S1 for primer information. The correct alignment of the gene was confirmed by partial sequencing of the plasmid using ACpBg-F/ACpSi-/R primers (Ali et al., 2013).

### Generation and Selection of Fungal Mutants

*Fusarium oxysporum* strain 11C was transformed with pSilent-1-FoPTR2 and pBARGPE1-FoPTR2 in order to respectively silence and overexpress the *FoPTR2* gene. Protoplasts were produced from fungal spores as previously described in Ali et al. (2013) and transformed with pSilent-1-FoPTR2 and pBARGPE1-FoPTR2, as described by Doohan et al. (1998). Putative silencing transformants were selected on PDA (Oxoid, United Kingdom) containing 60 μg ml^-1^ hygromycin (Sigma, Germany) (Doohan et al., 1998), whereas overexpression transformants were selected on minimal medium (Leung et al., 1995) containing 1000 μg ml^-1^ phosphinothricin (Sigma, Germany). Transformant stability was assessed (following successive subcultures) as previously described by Ali et al. (2013). Single spore colonies from the putative transformants were stored in 15% (vv^-1^) glycerol solution at –70°C. Transformation and plasmid integration was confirmed by both PCR and southern blot analysis. The PCR target/Southern blot probe was a fragment of the *hyg* gene for silencing mutants and of the *bar* gene for overexpression mutants. For genomic DNA extraction, mutant/wild type *F. oxysporum* was cultured in potato dextose broth (PDB; Oxoid, United Kingdom) and mycelium were harvested by centrifugation at 3200 rcf for 15 min and flash-frozen in liquid nitrogen followed by freeze-drying. Dried mycelium was ground into fine powder in a mixer mill (Retsch MM400, Germany) at 30 Hz for 1 min with two 2.3 mm steel beads and total genomic DNA was isolated by adding fungal DNA extraction buffer as described by Edel et al. (2000). DNA was purified by phenol-chloroform treatment and subjected to ethanol precipitation and re-suspended in Tris–EDTA buffer (pH 7.4). For putative silencing mutants, PCR was conducted using *hyg*-specific primers (Hyg-F1/R1; see Supplementary Table S1), which were used to amplify a 747 bp *hyg* gene fragment from either 100 ng gDNA of putative mutants or 50 ng pSilent-1 plasmid DNA (as a positive control and to produce probe for southern blot analysis). For the putative overexpression mutants, PCR was conducted using *bar*-specific primers (Bar-F1/R1; See Supplementary Table S1), which were used to amplify a 433 bp *bar* gene fragment from 100 ng gDNA of putative mutants or 50 ng pBARGPE1 plasmid DNA (as a positive control and to produce probe for southern blot analysis). The PCR reaction components and the conditions were as described above. For visualization of PCR amplicons, 10 μl of PCR product were subjected to electrophoresis in 1% (w v^-1^) Tris Acetate-EDTA (TAE) buffer agarose gels containing 0.5 μg ml^-1^ ethidium bromide, and visualized using Imagemaster VDS and Liscap software (Pharmacia Biotech, United States).

For Southern blot analysis of mutants, genomic DNA (10 μg) from wild type *F. oxysporum* strain 11C and putative mutants was digested overnight with restriction enzymes (New England Biolabs, United States). Blots of putative gene-silenced mutants were prepared using DNA digested with either *SacI* (single digest of the *hyg* gene) or *SacI* plus *KpnI* (double digest of the *hyg* gene). Likewise, blots of putative overexpression mutants were prepared using DNA digested with either *SmaI* (single digest of the *bar* gene) or *SmaI* plus *PmlI* (double digest of the *bar* gene. Digests were electrophoresed through 0.8% (wt vol^-1^) TAE buffer agarose gels (at 30 v; overnight) and blotted onto reinforced nitrocellulose membrane (Optitran BA-S85, Schleich and Schuell, Germany), as described by Sambrook et al. (2001). A 747 bp fragment of the *hyg* gene and a 433 bp fragment of the *bar* gene (amplified by PCR as described above) were respectively used as probes for Southern blot analysis of putative gene silenced and overexpressing mutants. The AlkPhos Direct Labeling and Detection System with CDP-Star (GE Healthcare, United States) were used for labeling and detecting the *hyg* and *bar* probes. The hybridization and detection procedures were performed according to the manufacturer’s protocol.

### Statistical Analysis

All real-time RT-PCR data and bioethanol yield data from the hexose, pentose and wheat straw experiments featuring mutants silenced in the *FoPTR2* function were non-normally distributed, as determined using the Ryan Joiner test (Ryan and Joiner, 1983) within Minitab (Minitab release©16, 2000 Minitab Inc.). All except the temporal analysis of *FoPTR2* transcript accumulation, bioethanol yield data from the sugar and wheat straw experiments could be transformed to fit a normal distribution using the Johnson transformation (Ryan and Joiner, 1983) within Minitab (Minitab release©16, 2000 Minitab Inc.). All other data sets were normally distributed. The homogeneity of data sets across replicate experiments was confirmed by two-tailed correlation analysis (non-normal data: Spearman Rank; normal data: Pearson product moment) conducted within the Statistical Package for the Social Sciences (SPSS 11.0, SPSS Inc.; *r* ≥ 0.828; *P* = 0.05) (Snedecor and Cochran, 1980). Therefore, data sets from the replicate experiments were pooled for further statistical analysis. The Statistical Package for the Social Sciences (SPSS 11.0, SPSS Inc.) was used to analyze the significance of treatment effects using one-way ANOVA with *post hoc* pair wise Tukey’s procedure comparisons (*P* = 0.05; for normally distributed data), or the Kruskal–Wallis H test (for non-normally distributed data) (Snedecor and Cochran, 1980). Pearson product moment analysis was used to determine the correlation between mean values from different normally distributed data sets.

## Results

### Cloning and Characterization of the *F. oxysporum FoPTR2* Gene

**Figure 1:**
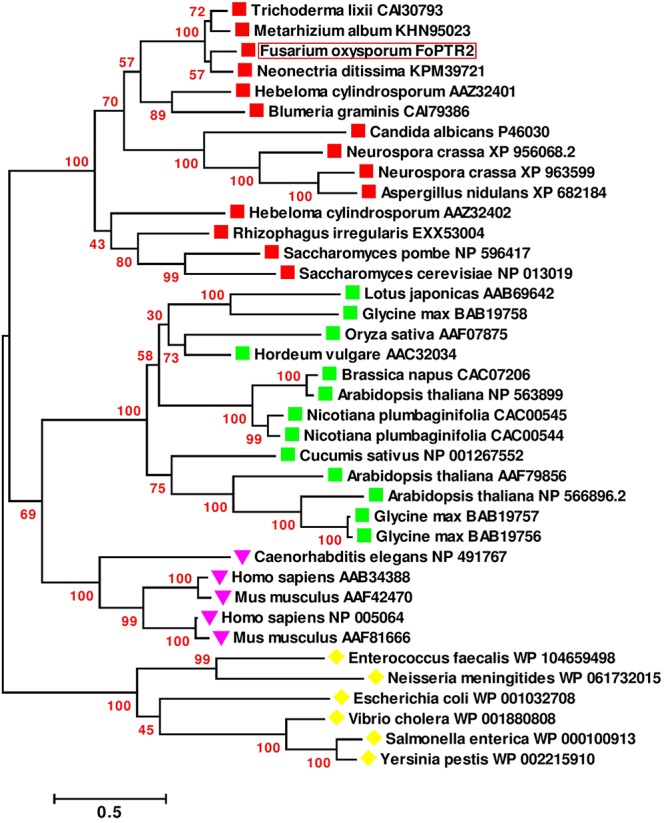
Phylogenetic tree illustrating the sequence analogy among the peptide transporter (PTR) proteins from *Fusarium oxysporum* and 37 other PTR proteins from fungi, mammals, plants and bacteria. 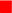 Indicates fungal/yeast peptide transporters; 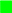 indicates plant peptide transporters; 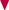 indicates human/animal peptide transporters; 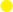 indicates peptide transporters from bacteria. Sequences are annotated with the NCBI *GenBank* Identifier numbers, except for the deduced *F. oxysporum FoPTR2* protein. Amino acid sequences were aligned via the CLUSTALW algorithm (Thompson et al., 1994) and evolutionary relationship was inferred using the maximum-likelihood algorithm with bootstrap (1000 replicates). Branch length represents the number of amino acid substitutions per sequence. Branches are labeled with bootstrap values. All positions containing gaps and missing data were eliminated. There were a total of 88 positions in the final dataset. Evolutionary analyses were carried out in MEGA6 (Tamura et al., 2013).

Based on sequence analysis of the full-length *FoPTR2* mRNA, the ORF was determined to be 1473 nucleotides (GenBank accession no. KX922691). NCBI domain analysis indicated that the protein contains a major facilitator superfamily (MFS) conserved domain and a proton-dependent oligopeptide transport domain. The crystal structure of the deduced amino acid sequence highlighted 11 helixes (Supplementary Figure S3A). Domain analysis predicted 11 transmembrane helices with a short N-terminal end protruding out of the cytoplasm and the C-terminal end protruding into the cytoplasm (Supplementary Figure S3B). The deduced amino acid sequences showed similarity (>80%) with five peptide transporters (FOXG_15681, FOXG_01591, FOXG_09811, FOXG_12388 and FOXG_04876) encoded within the *F. oxysporum* f. sp. *lycopersici* (strain 4287) genome. A global sequence similarity search of the *F. oxysporum FoPTR2* protein sequence against the NCBI protein dataset indicated that the homologs of this protein are only present in filamentous fungi. A phylogenetic tree placed *FoPTR2* amongst a subgroup composed of filamentous fungi and yeast PTRs (Figure 1). Of these, *FoPTR2* showed highest homology to *Neonectria ditissima PTR2* (76%).

### Temporal Accumulation of *FoPtr2* During CBP

**Figure 2:**
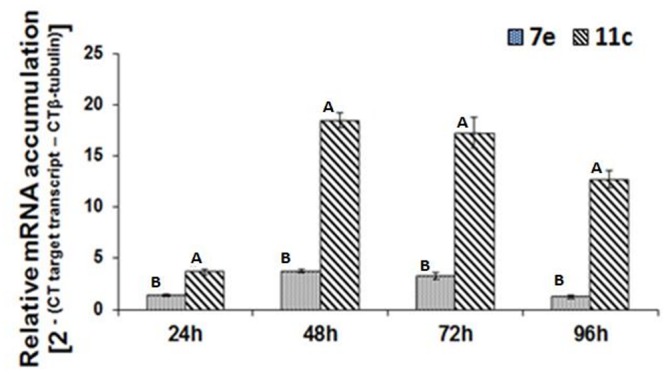
Temporal analysis of peptide transporter (*FoPTR2*) transcript accumulation throughout the saccharification of wheat straw/bran by *F. oxysporum* strains 11C and 7E. *F. oxysporum* was aerobically cultured on a wheat straw/bran mix (10:1 ratio) and RT-PCR was performed utilizing RNA samples collected at either 24, 48, 72 or 96 h post-inoculation. *FoPTR2* transcript expression levels were calculated in comparison with that of the housekeeping gene β-tubulin (Ali et al., 2013). Results were based on two replicated trials, each with three replicates per time point per strain. Bars specify SEM. For any given time point, columns with different letters are significantly different (*P* ≤ 0.05).

Real-time RT-PCR was used to analyze the temporal accumulation of the *FoPTR2* transcript in *F. oxysporum* strain 11C and 7E during the aerobic growth phase, relative to β-tubulin (housekeeping gene) (Figure 2). In both strains, transcript levels peaked at 48–72 h post-inoculation. As determined by real-time RT-PCR analysis, *FoPTR2* transcription was highly up-regulated in *F. oxysporum* strain 11C as compared to 7E, at all times (*P* ≤ 0.05) (Figure 2). Expression in strain 11C varied from 2.8 to 10 fold higher than in strain 7E (at 24 and 96 h, respectively).

### Effect of Nitrogen and Glucose on *FoPTR2* Transcription

**Figure 3:**
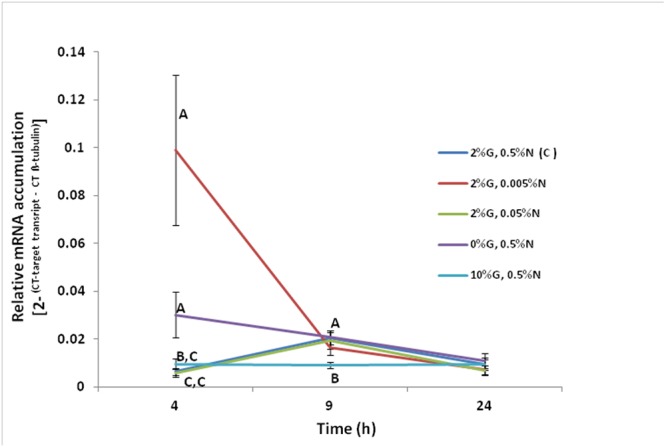
Influence of nitrogen and glucose on the transcription of the *F. oxysporum FoPTR2* gene. Aerobic shake flask cultures of *F. oxysporum* strain 11C were cultured in minimal media (Penttila et al., 1987). Ammonium sulfate (NH_4_SO_4_ – N source) and glucose concentrations were manipulated to mimic nitrogen starvation and glucose rich/limited conditions in separate cultures. The control treatment was 2%G, 0.5%N, while the other treatments were as follows: (i) 2%G, 0.005%N; (ii) 2%G, 0.05%N; (iii) 0%G, 0.5%N; (iv) 10%G, 0.5%N. Mycelia was harvested at either 4, 9 or 24 h post-inoculation of flasks and RNA was used for gene expression studies. *FoPTR2* transcript accumulation was measured in comparison to that of the housekeeping gene β-*tubulin* (Ali et al., 2013). Results are based on three replicated trials, each of which included three flasks per medium per time point per treatment. Bars specify SEM. For any given time point, columns with different letters are significantly different (*P* ≤ 0.05).

Experiments were carried out in order to ascertain if nitrogen and/or glucose regulate the transcription of the *F. oxysporum FoPTR2* gene. Gene up-regulation by both glucose and nitrogen starvation was observed at 4 h post-inoculation. The *FoPTR2* mRNA levels were highest at 4 h when the fungus was grown in media containing 100-fold less nitrogen (2%G, 0.005%N) as compared to other media where nitrogen was available or glucose was either limited or elevated (Figure 3). *FoPTR2* expression was also elevated, to a lesser extent at 4 h in medium lacking glucose (0%G, 0.5%N) and was repressed at both 4 and 9 h in medium containing excess glucose (10%G, 0.5%N), relative to the control medium.

### Gene Silenced and Overexpression Mutants

**Figure 4:**
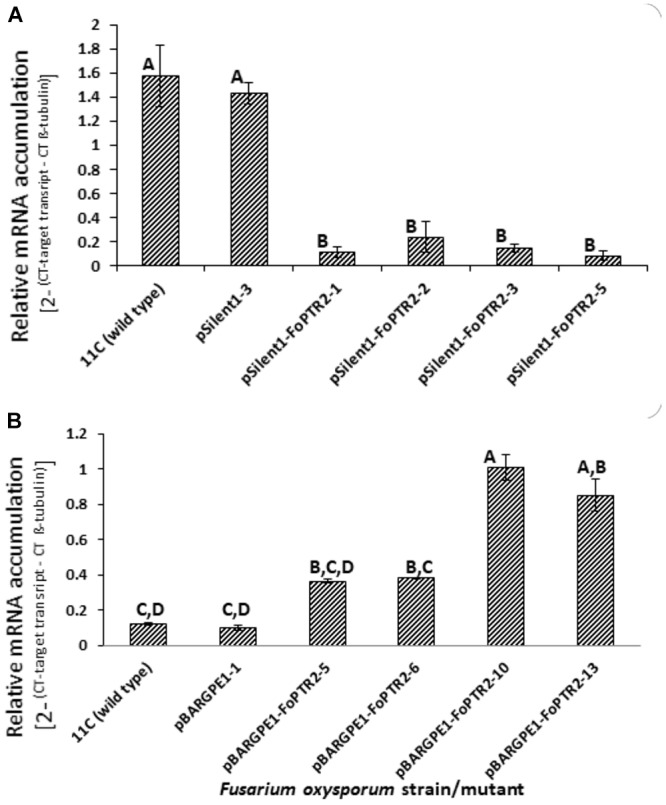
Relative expression of peptide transporter (*FoPTR2*) in wild type and gene-silenced/overexpressed mutants of *F. oxysporum* strain 11C. **(A)** Gene silenced mutants pSilent-1-FoPTR2-1, 2, 3, 5, mutant pSilent1-1 transformed with the empty silencing vector and wild type fungus; **(B)** gene overexpressed mutants pBARGPE1-FoPTR2-5, 6, 10 and 13, mutant pBARGPE1-1 transformed with the empty over expression vector and wild type fungus. In the case of both A and B, fungal cultures were grown aerobically on a straw/bran (10:1 ratio) mix for 48 h. *FoPTR2* transcript accumulation in RNA extracts was measured in comparison to that of the housekeeping gene β-*tubulin* (Ali et al., 2013). Two experiments were conducted, each with three replicates per treatment. Bars specify SEM. Columns with different letters are significantly different (*P* ≤ 0.05).

Both overexpressing and gene silenced mutants were produced for *FoPTR2* in *F. oxysporum* strain 11C. Transformation with the empty vector pSilent-1 or with pSilent-1-FoPTR2 was confirmed by PCR analysis of the *hyg* gene (Supplementary Figure S4A) and by Southern blot analysis using a PCR-amplified segment of the *hyg* gene as a probe (Supplementary Figure S4B). Southern analysis deduced that the four silencing mutants (pSilent-1-FoPTR2-1, pSilent-1-FoPTR2-2, pSilent-1-FoPTR2-3 and pSilent-1-FoPTR2-5) contained a single vector copy integration. Transformation with empty overexpression vector pBARGPE1 or with pBARGPE1-FoPTR2 was confirmed by PCR analysis of the *bar* gene (Supplementary Figure S5A). Southern blot analysis using a PCR-amplified segment of the *bar* gene as a probe verified that mutants pBARGPE1-FoPTR2-5, pBARGPE1-FoPTR2-6, pBARGPE1-FoPTR2-10 and pBARGPE1-FoPTR2-13 have a single copy of the vector integrated into the genomic DNA (Supplementary Figure S5B). Real-time RT-PCR analysis of *FoPTR2* transcript levels in fungi cultured for 24 h on straw/bran under aerobic conditions confirmed the efficacy of gene silencing and overexpression (Figure 4). There was almost a 3-fold decrease in the expression level of this gene in the three *FoPTR2*-silenced mutants (pSilent-1-FoPTR2-1, pSilent-1-FoPTR2-3 and pSilent-1-*FoPTR2*-5) as compared to the wild type strain 11C and the mutant (pSilent-1-1) transformed with the empty vector (Figure 4A). For the overexpression mutants, compared to the wild type or the empty vector (pBARGPE1-1) mutant, transcript levels in pBARGPE1-FoPTR2-10 and pBARGPE1-FoPTR2-13 were significantly higher (more than 2-fold higher; *P <* 0.05) (Figure 4B). Silencing mutants pSilent-1-FoPTR2-3 and pSilent-1-*FoPTR2*-5, and overexpression mutant pBARGPE1-FoPTR2-13 were used for subsequent experiments (unfortunately, pBARGPE1-FoPTR2-10 was lost due to contamination).

### Role of the *FoPTR2* Gene During Lignocellulose Bioconversion by *F. oxysporum*

**Figure 5:**
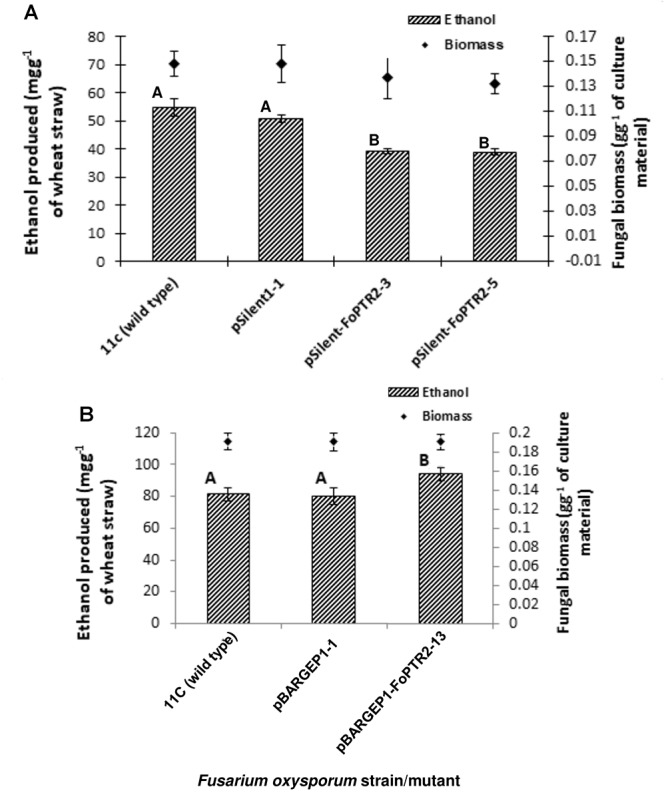
Silencing and overexpressing the peptide transporter (*FoPTR2*) gene and its effect on *F. oxysporum* strain 11C’s capacity to colonize and produce bioethanol from a wheat straw/bran mix (10:1 ratio). **(A)** Gene silenced mutants pSilent-1-FoPTR2-3 and 5 mutant pSilent-1-1 transformed with the empty silencing vector and wild type fungus; **(B)** gene overexpressed mutant pBARGPE1-FoPTR2-13, mutant pBARGPE1-1 transformed with the empty overexpression vector and wild type fungus. For both A and B, fungi were cultured on wheat straw/bran for 96 h of aerobic growth and succeeded by 96 h of oxygen-limiting growth. The yield of bioethanol was quantified using QuantiChromTM Ethanol Assay Kit (DIET-500; BioAssay Systems, United States). Bars specify SEM. Columns with different letters are significantly different (*P* ≤ 0.05).

Silencing of *FoPTR2* resulted in significant reductions in the amount of bioethanol yielded by strain 11C following CBP of a straw/bran mix (*P* ≤ 0.05) (Figure 5). The two silencing mutants tested, pSilent-1-FoPTR2-3 and pSilent-1-FoPTR2-5, respectively yielded 16.8 and 18.8% less ethanol from straw/bran as compared to a mutant strain transformed with the empty vector (*P* ≤ 0.05). There was a correlation between the level of *FoPTR2* transcript accumulation and bioethanol productivity (*r* = 0.942; *n* = 12; *P* ≤ 0.05). Overexpression of the *FoPTR2* gene increased the ethanol yielded from a straw/bran mix by almost 17% in the case of the overexpression mutant pBARGPE1-FoPTR2-13, as compared to the control transformed with the empty vector (Figure 5B) and showed a statistically significant increase in ethanol yield (*P* ≤ 0.05). There was a significant correlation between the level of transcript accumulation and ethanol production (*r* = 0.801; *n* = 9; *P* ≤ 0.05). Neither gene silencing nor overexpression affected the fungal biomass produced (*P* ≥ 0.05) (Figure 5).

### Effect of *FoPTR2* on Bioethanol Yield From C5 and C6 Sugars

**Figure 6:**
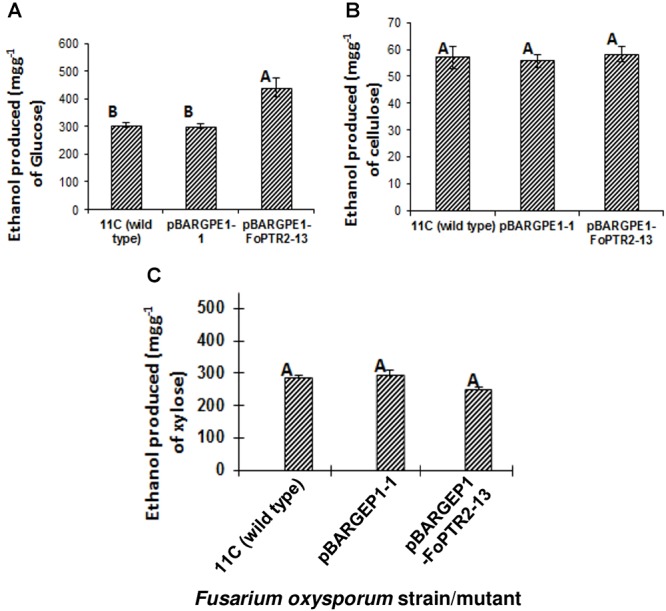
Overexpressing the peptide transporter (*FoPTR2*) and its influence on the capacity of *F. oxysporum* strain 11C to ferment hexose and pentose sugars to bioethanol. Gene overexpressed mutant pBARGPE1-*FoPTR2*-13, mutant pBARGPE1-1 transformed with the empty over expression vector and wild type fungus were tested. Fungi were cultured in minimal media shake flask cultures supplemented with **(A)** 1% (w/v) glucose, **(B)** cellulose and **(C)** xylose for 24 h aerobic followed by 96 h oxygen-limited growth. Bars specify SEM. Columns with different letters are significantly different (*P* ≤0.05).

The influence of the *FoPTR2* on the ability of *F. oxysporum* to ferment hexose and pentose sugars to bioethanol was evaluated by comparing the capacity of the overexpressing mutant pBARGPE1-*FoPTR2*-13, relative to wild type fungus and control mutants transformed with the empty vectors. *FoPTR2* silenced mutants had no significant effect on glucose fermentation (*P* ≥ 0.05; result not shown). The overexpression mutant tested produced significantly more (45% more) bioethanol from glucose (*P* ≤ 0.05) than did either the empty vector transformant or the wild type fungus (Figure 6A). But it did not yield more ethanol from either cellulose (*P* ≥ 0.05) (Figure 6B) or xylose (*P* ≥ 0.05) (Figure 6C).

## Discussion

The positive contribution of *FoPTR2* toward the fungal capacity to produce bioethanol from a straw/bran mix and from glucose affirms its role in the CBP process and highlights it as a target for the enhancement of fungal CBP. This gene was activated throughout the aerobic phase of CBP, a hallmark of most membrane proteins whose expression level is usually quite low and can only be detected during the log phase of growth (Schertler, 1992). The conserved domain and transmembrane (TM) domain analysis of the deduced amino acid sequence highlighted 11 helixes indicative of the proton-dependent oligopeptide transporters (PTR/POT) class of transporters belonging to the much larger MFS of secondary active transport proteins (Pao et al., 1998; Reddy et al., 2012). The *FoPTR2* gene is 1473 bp and encodes a protein of 490 amino acids with a predicted molecular mass of 54.65 kDa. Computer-assisted analysis of the *FoPTR2* protein sequence identified the presence of 2/3 of the PTR-signature motifs including the consensus sequence “GTGGIKPXV” spanning the end of the fourth domain (Meredith and Boyd, 2000) and the “FYING” motif located in the fifth TM domain (Steiner et al., 1995).

Phylogenetic analysis of amino acid sequences of the PTR family from plants, bacteria, animals and yeast placed the *FoPTR2* sequence amongst a cluster of other fungal PTRs, highlighting *FoPTR2’*s fungal ancestry. Given the high homology to *Trichoderma sp.* peptide transporters *ThPTR2* (Vizcaíno et al., 2006) and TrEST-A0793 (Chambergo et al., 2002), we hypothesized that the *FoPTR2* activity may be affected by nitrogen and possibly glucose concentrations. As with *ThPTR2*, the expression profile of *FoPTR2* increased exponentially in low concentrations of ammonium sulfate after 4 h of incubation while glucose-free conditions also appeared to partially stimulate *FoPTR2* activity in comparison to expression data from the complete medium. As outlined earlier, the PTR/POT system in yeasts is believed to be strictly governed by NCR, as its’ di/tri-peptide transport activity is regulated by the quality of the nitrogen source available (Perry et al., 1993; Basrai et al., 1995). NCR is a nutrient sensing mechanism that detects external N concentrations and dictates the appropriate transcriptional response and has been analyzed in detail in filamentous ascomycetes (Jarai and Buxton, 1994; Marzluf, 1997) and specifically *F. oxysporum* (Divon et al., 2006). Fungi can utilize a wide range of nitrogen sources, however, glutamine and ammonium are favored by *Fusarium sp.* and both are known to trigger NCR (Divon et al., 2006). Genes encoding peptide transporters have previously been identified to be among a specialized subset of genes up-regulated by *F. oxysporum* to enhance fungal fitness in nitrogen-poor environments (Divon et al., 2005). It seems clear that *FoPTR2*, similar to other PTR2s is under the control of a system sensing extracellular organic N concentrations. Gene expression data presented here has confirmed *FoPTR2* is highly activated in nitrogen-poor environments, most likely as a means to source and mobilize alternative nitrogen sources.

In the case of glucose repression (carbon catabolite repression (CCR), *ThPTR2* was considered not to be repressible by glucose, in contrast to *TrESt-A0793* (Chambergo et al., 2002; Vizcaíno et al., 2006). Our investigations showed a partial stimulation of *FoPTR2* expression after 4 h incubation in glucose-free medium, indicating a possible alleviation of CCR comparable to that reported previously for *ThPTR2* (Vizcaíno et al., 2006) and *TrESt-A0793* (Chambergo et al., 2002). Although CCR may have been mitigated, the presence of a rich nitrogen source (0.5% ammonium sulfate) in the glucose-free media may have subdued the transcription of *FoPTR2*. Furthermore, NCR appears to be dominant over CCR in its control of *FoPTR2*, as was the case *ThPTR2* in *T. harzanium* (Vizcaíno et al., 2006). In summary, the expression profiles recorded here for *FoPTR2* are analogous to those previously reported for other fungal PTR genes whilst further supporting the assertion that the encoded *FoPTR2* protein is indeed a PTR class peptide transporter.

During *Fusarium*-mediated CBP of untreated wheat straw/bran, the rate of decomposition of untreated straw/bran is slow and nutritional challenges such as nitrogen limitation are common. An inadequate supply of nitrogen inhibits fungal fitness and is a limiting factor in fungal pathogenesis (Snoeijers et al., 2000; Divon et al., 2006). Following fungal degradation of organic matter, the end products of this degradation are transported across the cellular membrane. Imported peptides can be promptly hydrolyzed by peptidases and recycled as sources of amino acids, N or carbon. The quality of the nitrogen source that becomes available and its assimilability is known to have a profound effect on microbial growth, fermentation and subsequent ethanol productivity (Slininger et al., 2006; Laopaiboon et al., 2009) and particularly in *F. oxysporum* (Panagiotou et al., 2006). Silencing of the *FoPTR2* gene reduces the number of peptide transporters in the cell membrane, thus reducing the symport of external nitrogenous peptides. As the nitrogen status of the cell impacts many cellular processes, thwarting the ability of these mutants to accumulate nitrogen may have affected the overall functionality of *F. oxysporum* and its ability to carry out CBP. Conversely, overexpression mutant should theoretically have a greater ability to mobilize peptides, conferring an advantage in their infection and indirectly, their bioconversion competency. The overexpression mutant pBARGPE1-FoPTR2-13 showed both a significant increase in gene expression and in ethanol yield (17% increase; *P* ≤ 0.05) as compared to the control empty vector mutant. This is quite significant in terms of technology improvement and the economic advantage if the same can be achieve on an industrial scale. The negative impact on ethanol yield when *FoPTR2* was silenced affirms *FoPTR2*’s involvement and importance in the CBP process.

Glucose is the most abundant sugar found in plant biomass and is preferentially utilized by microorganisms. Overexpression of the *FoPTR2* protein in *F. oxysporum* conferred mutant with an increased capacity to ferment ethanol from glucose. This result may seem puzzling considering PTRs are reportedly suppressed by glucose through CCR. Some PTR transporters from plants (*Arabidopsis* – (*AtNRT1*); (Rentsch et al., 1995) and fungi (*Hebeloma cylindrosporum* – (*HcPTR2A)*; Benjdia et al., 2005) are activated in the presence of nitrate and exhibit a low-affinity transport for nitrate. Similarly, the presence of nitrate in the minimal media used in this assay may have evoked *FoPTR2* activity. Fungal nitrate metabolism is an energy yielding process that generates intracellular NADH (Takasaki et al., 2004). Given NADH’s key role as a cofactor to enzymes involved in the fermentation process, a higher proportion of available NADH may have improved the rate of glucose oxidation to ethanol. In particular, the final step in the fermentation of glucose requires NADH as a cofactor for alcohol dehydrogenase (ADH), the enzyme that converts acetaldehyde to ethanol. Thus the rate of assimilation of nitrogen in *F. oxysporum* would appear to play a critical role in the microbe’s fermentative capacity. Conversely, there was no improvement in ethanol yields from cellulose and xylose.

In conclusion, this study confirmed that the CBP of lignocellulose by *F. oxysporum* is a highly intricate system encompassing the activity of genes that are not directly related to saccharification or fermentation. The *FoPTR2* identified in this study is specific to filamentous fungi and clearly influenced the CBP efficiency of *F. oxysporum*. Like most PTR proteins characterized in other organisms, *FoPTR2* is governed by the concentration of nitrogen in the medium. The ability to respond and assimilate alternate nitrogen sources in nitrogen-poor conditions is a prerequisite for successful colonization of plants by *Fusarium* and *FoPTR2* would appear to be involved in this response. This nutritional versatility maintains fungal fitness allowing it to hydrolyze lignocellulosic substrates with greater efficiency. As manipulation of the *FoPTR2* gene influences CBP activity of *F. oxysporum*, it becomes evident that this can be a target gene for use in the development of any highly efficient engineered bioprocessing agent.

## Author Contributions

SA, FD, and BN conceived and designed the experiments. SA and BN performed the experiments and wrote the manuscript. SA and FD analyzed the data. FD and EM contributed reagents, materials and analysis tools. SA, EM, and FD provided intellectual and editorial comments.

## Conflict of Interest Statement

The authors declare that the research was conducted in the absence of any commercial or financial relationships that could be construed as a potential conflict of interest.

## Footnotes

1 http://www.ncbi.nlm.nih.gov/projects/gorf/

2 http://www.ncbi.nlm.nih.gov/Structure/cdd/wrpsb.cgi

3 http://www.cbs.dtu.dk/services/TMHMM/

4 http://swissmodel.expasy.org

5 http://www.ebi.ac.uk/Tools/msa/clustalo/
