# Supplementary material for: A Major Facilitator Superfamily Peptide Transporter From Fusarium oxysporum Influences Bioethanol Production From Lignocellulosic Material
Source: Front Microbiol. 2019 Feb 26;10:295. doi: 10.3389/fmicb.2019.00295 (PMC6399157; doi:10.3389/fmicb.2019.00295)
Supplement: Supplementary file 1 [file Data_Sheet_1.DOCX]

**Supplementary information**

**A Major Facilitator Superfamily Peptide Transporter from *Fusarium oxysporum* influences bioethanol production from lignocellulosic material.**

**Brian Nugent^1^, Shahin S. Ali^1 ,$,*^, Ewen Mullins^2^ and Fiona M. Doohan^1^**

^1^Molecular Plant-Microbe Interactions Laboratory, School of Biology and Environmental Science, University College Dublin, Dublin 4, Ireland

^2^Department of Crop Science, Teagasc Research Centre, Oak Park, Carlow, Ireland

^$^Current address: SPCL, USDA/ARS Beltsville Agricultural Research Center, MD 20705, USA

*Corresponding author Email: [shahinsharif.ali@gmail.com](mailto:shahinsharif.ali@gmail.com)

**Supplementary figures**


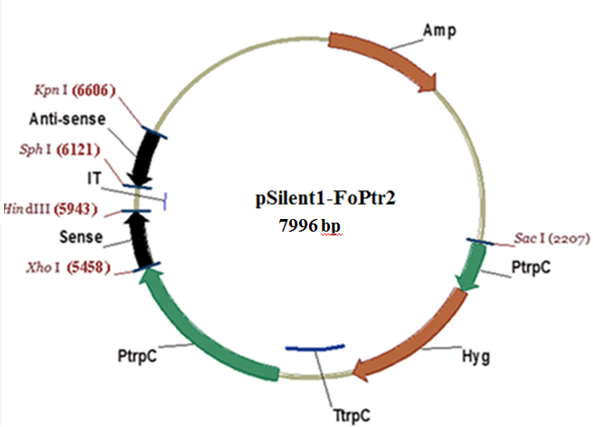


**Figure S1** The silencing vector pSilent-1-*FoPTr2* was constructed using the pSilent-1 vector (Nakayashiki et al., [2005](#_ENREF_3)) which contains Aspergillus nidulans *trp*C promoter and terminator flanking two MCS separated by an intron from a Magnaporthe grisea cutinase gene. A 485bp fragment of the *FoPTR2* gene with appropriate overhanging restriction sites (as shown in the figure) was inserted into each of the two MCS in the sense (upstream of the intron) or antisense (downstream of the intron) direction. Vector diagram was generated using Vector NTI Advance® 11.5 software (Invitrogen).


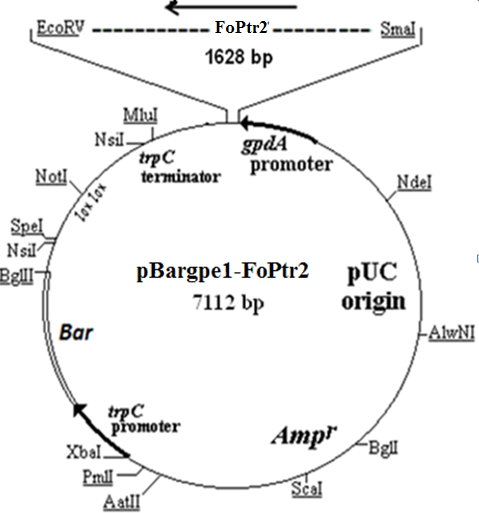


**Figure S2** The over expression vector pBARGPE1-PepT was constructed using the pBARGPE1 vector (Pall and Brunelli 1993) which contains Aspergillus nidulans *gpdA* promoter and *trpC* terminator flanking a MCS and bar as a selectable marker gene which provides resistance to basta or phosphinothricin. The 1628bp full-length sequence of the *PepT* gene with appropriate overhanging restriction sites was inserted into the MCS (as shown in the figure).


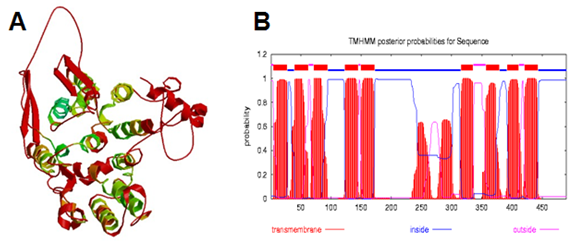


**Figure S3** Putative crystal structure and transmembrane domains (TMD) of the *Fusarium oxysporum* peptide transporter (*FoPTR2*) and sequence similarity with other PTR proteins from *F. oxysporum*. **(A)** The putative crystal structure was based on a template of a proton dependent oligopeptide (POT) family transporter 2xut.2 (Newstead et al.*,* 2011) (3.62 A) (*E*value 4.2E-45). Colour indicates residue error; QMEAN Z-Score: -12.225 (Benkert et al., 2011). The putative crystal structure was generated using SWISS-MODEL Workspace (http://swissmodel.expasy.org)(Arnold et al., 2006). **(B)** The TMDs were predicted with the TMHMM Server version 2.0 (http://www.cbs.dtu.dk/services/TMHMM/).

**Figure S4** Confirmation of uptake and genomic integration of *FoPTR2* gene silencing plasmid pSilent-1-FoPTR2 into *Fusarium oxysporum*. (A) PCR was used to confirm if fungal mutant genomic DNA extracts contained the hygromycin (*hyg*) gene from the pSilent1 backbone. Lanes: 1, no DNA (negative control); 2-6, gDNA from mutants pSilent-1-FoPTR2-1, 2, 3, 4 & 5 transformed with the *FoPTR2*-silencing vector; 7 & 8 gDNA from mutant pSilent-1-A & B transformed with the empty silencing vector; 9, gDNA from wild type fungus strain 11C; 10, plasmid pSilent1 DNA (positive control). Arrow indicates *hyg* PCR product (747bp). (B) Southern blot analysis was used to confirm plasmid integration in *SacI* digested fungal genomic DNA and to confirm gene copy number, using a 747nt fragment of the *hyg* gene as a probe. Lanes 1 – 6 represent DNA digested with *SacI*. Lane 1, gDNA from wild type fungus strain 11C; Lane 2, gDNA from mutant pSilent-1-A transformed with the empty silencing vector; 3 – 6, gDNA from mutant’s pSilent-1-FoPTR2-1, pSilent-1-FoPTR2-2, pSilent-1-FoPTR2-3 and pSilent-1-FoPTR2-5. Arrows indicate molecular size (Kb) based on the 1kb DNA ladder (Solis BioDyne, Estonia).

**Figure S5** Confirmation of uptake and genomic integration of *FoPTR2* gene overexpression plasmid pBARGPE1-FoPTR2 into *Fusarium oxysporum*. (A) PCR was used to confirm if fungal mutant genomic DNA extracts contained the *bar* gene from the pBARGPE1 backbone. Lanes represent: 1 to 4, gDNA from mutants pBARGPE1-FoPTR2-5, 6, 10 & 13 transformed with the *FoPTR2* -over expression vector; 5 & 6, gDNA from mutant pBARGPE1-1 & 2 transformed with the empty over expression vector; 7, plasmid pBARGPE1 DNA (positive control); 8, gDNA from wild type fungus strain 11C; 9 no DNA (negative control). Arrow indicates *bar* PCR product (433bp). (B) Southern blot analysis was used to confirm plasmid integration in *SmaI* digested fungal genomic DNA and to determine gene copy number, using a 433bp fragment of the *bar* gene as a probe. Lanes 1 – 6 represent DNA digested with *SmaI*; Lane 1, gDNA from mutant pBARGPE1-1 transformed with the empty over expression vector (digested with *SmaI*); Lanes 2-5, gDNA from mutants pBARGPE1-FoPTR2-5, pBARGPE1-FoPTR2-6, pBARGPE1-FoPTR2-10 and pBARGPE1-FoPTR2-13; Lane 6, gDNA from wild type fungus strain 11C; Arrows indicate molecular size (Kb) based on the 1kb DNA ladder (Solis BioDyne, Estonia).

**Supplementary table**

**Table S1 Primers used in this study**

| Primer ID | Target gene | Forward Primer(5’ – 3’) | Reverse primer (5’ – 3’) |
| --- | --- | --- | --- |
| PepT-F1/R1 | *FoPTR2* | ATGGGACCCAAGGCTGCG | TCACATCTTCTCAATGTCCCT |
| RACE-PepT-MF/MR | *FoPTR2* | GTCGATGAGGTCCGTCGTG | GGGCCTTGGAGTAGGCGTACTC |
| ß-tub-F/R | ß-tubulin | CAACAACATCCAAACAGCC | CTCACCAACACGCTTGAAGA |
| Hyg-F1/R1 | *Hyg* | TTCCGGAAGTGCTTGACATT | TTCTACACAGCCATCGGTCC |
| ACpSi-F/R | *trpC* promoter/terminator | ACGACCCGGTCATACCTTCT | AAACAGCTTGACGAATCTGGA |
| Si_PepT-L-F2/R2 | *FoPTR2* | CCCTCGAGGGTCGATGAGGTCCGTCGTG | AAAGCTTGGGGCCTTGGAGTAGGCGTACTC |
| Si_PepT-R-F2/R2 | *FoPTR2* | GGGGTACCGTCGATGAGGTCCGTCGTG | GGCATGCCGGGCCTTGGAGTAGGCGTACTC |
| FL_PepT-F/R | *FoPTR2* | CGGATATCGATGGGACCCAAGGCTGCG | CCCCCGGGGGTCACATCTTCTCAATGTCCCT |
| ACpBg-F | *gpdA* promoter | TCAGTTCGAGCTTTCCCACT | TCACATCTTCTCAATGTCCCTAG |
| Bar-F1/R1 | *bar* | GCACCATATCGTCAACCACTACATCG | AGCTGCCAGAAACCACGTCATG |
| Primers were design using the Primer3 software (version 0.4.0; http://frodo.wi.mit.edu/primer3/). | | | |

**Reference:**

Arnold, K., Bordoli, L., Kopp, J., Schwede, T. (2006) The SWISS-MODEL workspace: a web-based environment for protein structure homology modelling. Bioinformatics 22: 195-201.

Benkert, P., Biasini, M., Schwede, T. (2011). Toward the estimation of the absolute quality of individual protein structure models. Bioinformatics 27: 343-350.

Nakayashiki, H., Hanada, S., Quoc, N.B., Kadotani, N., Tosa, Y., Mayama, S. (2005). RNA silencing as a tool for exploring gene function in ascomycete fungi. Fungal Genet. Biol. 42:275–283.

Newstead, S., Drew, D., Cameron, A.D., Postis, V.L.G., Xia, X., Fowler, P.W., Ingram, J.C., Carpenter, E.P., Sansom, M.S.P., Mcpherson, M.J., Baldwin, S.A., Iwata, S. (2011) Crystal structure of a prokaryotic homologue of the mammalian oligopeptide-proton symporters, PepT1 and PepT2. EMBO J. 30: 417–426.

Pall, M., Brunelli, J. (1993). A series of six compact fungal transformation vectors containing polylinkers with multiple unique restriction sites. Fungal Genet. Newsl. 40: 59–62.
